# Implications for Self-Management among African Caribbean Adults with Noncommunicable Diseases and Mental Health Disorders: A Systematic Review

**DOI:** 10.3390/biomedicines10112735

**Published:** 2022-10-28

**Authors:** Cherlie Magny-Normilus, Saria Hassan, Julie Sanders, Catrina Longhurst, Christopher S. Lee, Corrine Y. Jurgens

**Affiliations:** 1 William F. Connell School of Nursing, Boston College, Chestnut Hill, MA 02467, USA; 2 Department of Medicine, Emory University, Atlanta, GA 30307, USA

**Keywords:** mental health, self-management, chronic illness, noncommunicable disease, diabetes, hypertension, cardiovascular conditions, African Caribbean, Caribbean, African descent, Haitians

## Abstract

Mental health problems are common among individuals suffering from chronic noncommunicable diseases (NCDs) such as type 2 diabetes mellitus and hypertension. Self-management is essential in preventing NCD progression. Mental health problems can impede the ability to self-manage one’s NCDs. The African Caribbean population in the United States suffers from a high burden of NCDs and has unique societal factors that alter disease management. This systematic review aimed to better understand the burden of mental health problems among African Caribbean adults with one or more NCDs and explore the association between mental health disorders and the level of control of NCDs. A literature search was conducted for original research documenting the prevalence of mental illnesses in individuals with NCDs. Data were descriptively summarized. Fourteen studies met inclusion criteria. Three themes emerged: (1) prevalence of comorbid mental health problems and chronic NCDs; (2) factors that mitigate or mediate the association between mental health problems and chronic NCDs—(a) factors influencing self-management; (b) association between mental health and NCD outcome studies focused on (b1) risk factors and (b2) protective factors; and (3) varied results. Chronic disease self-management and disease outcomes are influenced by mental problems and the association is mitigated by complex factors in the African Caribbean population.

## 1. Introduction

The Caribbean population has the highest mortality rate of chronic noncommunicable diseases (NCDs) in the Americas, where chronic diseases account for 75% of deaths [1,2]. This population is disproportionately affected by diabetes, with a prevalence ranging between 11 and 18% [1,2]. In the North American and Caribbean regions, diabetes accounts for 13.8% of deaths [3]. This mortality rate is expected to increase over time [3]. NCDs require adequate management to prevent adverse outcomes, including mortality. Such management methods are important for African Caribbean individuals, a population with elevated incidence and mortality rate of hypertension and type 2 diabetes [2,4]. African Caribbeans also have elevated rates of hypertension compared with people of European and West African descent, and these rates have been associated with increased stroke incidence [4,5]. The high mortality rate associated with chronic diseases in the African Caribbean population underscores the need for further research on NCD management to create workable strategies for promoting better health.

Chronic disease self-management is the application of knowledge and skills required to complete daily disease management. Self-management strategies are essential to successful chronic disease treatments, as they are positively correlated with improved glycemic and hypertensive targets, reduction in complications, and quality of life improvements [6,7,8]. Exercise, healthy eating, and medication adherence are considered effective self-management strategies for hypertension and type 2 diabetes [7,8,9].

Engaging in effective self-management behaviors is often difficult when individuals living with NCDs have a comorbid mental health problem. African Caribbean adults have a higher prevalence and severity of major depressive disorder than their white counterparts [10,11]. However, in the United States (US), they are less likely to receive assistance for major depressive disorder than European Americans and African Americans [12]. It is worth noting that depressive disorders are the most significant contributor to years lived with disability in the Caribbean [10]. Moreover, African Caribbeans are more likely to report severe cases of anxiety than their African American and European American counterparts [13].

The relationship between control of mental health problems and control of NCDs is bidirectional. Poorly controlled mental health is associated with worse NCD outcomes, and NCDs and their management is associated with worsening mental health problems. Furthermore, poorly controlled mental health problems put individuals at higher risk of type 2 diabetes-related complications (e.g., retinopathy, nephropathy, neuropathy) and reduced quality of life [14,15]. Poor mental health is also associated with unsuccessful type 2 diabetes self-management behaviors, resulting in poor glycemic targets [14,16]. Individuals with heart disease have been found to have high rates of mood and anxiety disorders [17]. Comorbid depression, anxiety, and elevated stress among people with hypertension, is reported to reduce hypertensive medication adherence [16,18].

While there is substantial evidence to support these associations, there is a gap in the literature on how poorly controlled mental health problems are associated with the self-management of chronic diseases such as diabetes, hypertensiofxfn, hyperlipidemia, and cardiovascular disease in the African Caribbean population. Evidence is needed to inform interventions and practices that can address comorbid mental health and NCD management to improve the health and wellbeing of the African Caribbean population. Accordingly, the purpose of this study is to better understand the burden of mental health problems among African Caribbean adults with one or more NCDs. A secondary aim was to explore the association between mental health disorders and the level of control of NCDs. 

## 2. Materials and Methods

A systematic review of the literature was conducted and registered in PROSPERO: CRD42021269666. The Population, Intervention, Comparator, and Outcomes (PICO) model was applied to refine the research question [19]. The following terms were defined using the PICO framework:

- *Population*: African Caribbean adults with one or more NCDs, including type 2 diabetes, hypertension, hyperlipidemia, cardiovascular conditions.
- *Intervention*: Treatment for type 2 diabetes, hypertension, and hyperlipidemia, including exercise, healthy eating, blood glucose testing, and medication.
- *Comparator*: Usual care.
- *Outcomes*: Successful NCD self-management, successful mental health outcomes.

### 2.1. Search Strategy

The following databases were searched: Academic Search Premier, Cochrane, Google Scholar, PubMed, Science Direct, ProQuest, Web of Science, CINAHL, PsychInfo, Medline, Data Citation Index, ClinicalTrials.gov (accessed on 20 August 2022), and TRIP for full-text articles published in English. Search terms included African Caribbean, Caribbean, Eastern Caribbean, African Descent, Haitian, Mental Health, Depression, Psychosocial Factors, Diabetes, Diabetes Self-Management, Diabetes Distress, type 2 diabetes Risks, Cardiovascular Conditions, High Blood Pressure, Hypertension, Hyperlipidemia, and High Cholesterol. The primary search terms were consistently used for every database search.

All retrieved articles were assessed using the inclusion and exclusion criteria in Covidence. The inclusion criteria were studies of African Caribbean adults published in English with data collected on NCD outcomes based on objectively measured data and mental health outcomes assessed through self-reported questionnaires. The exclusion criteria included the following: other chronic health conditions (e.g., cancer) and all experimental studies on participants under 18 years of age, animal studies, and gestational diabetes. Two reviewers independently assessed each study in Covidence to determine if the inclusion criteria were met.

### 2.2. Data Extraction

The selected articles were summarized systematically using a data extraction table. Relevant details from each study, including author, purpose, design, location, samples, age, ethnicity, measures, and key findings, were extracted, and inputted in Table 1. The studied population was not categorized by gender, age, or other groups. Data analysis continued, comparing study results while focused on the purpose of the review. After data were independently extracted from each study by one of the authors (Catrina Longhurst, Cherlie Magny-Normilus, Julie Sanders), another author reviewed the analysis and critiqued the results to reach a consensus. This process was repeated for quality control and consistency.

### 2.3. Quality Appraisal/Risk of Bias

To assess risk of bias, the Cochrane Collaboration tool was used for a quality appraisal of experimental studies. Risk of bias was evaluated by three authors using the National Institutes of Health (NIH) Quality Assessment Tool [34] for observational and cross-sectional studies. All studies were assigned a yes, no, not applicable, or not reported to each of the 14 criteria, as outlined in the appraisal tool. Conversely, the Consolidated Criteria for Reporting Qualitative Research (COREQ) was used to determine the quality of qualitative studies [20]. Two reviewers independently labeled each article as good, fair, or poor, and answered questions from each of the COREQ’s three domains. Most of the selected articles were rated “good”, but one was rated “fair” due to the population sample. Any differences were deliberated in a group discussion until a consensus was reached.

### 2.4. Data Synthesis

Based on the inclusion and exclusion criteria, studies were selected. These studies were rated fair or good in the risk of bias assessment. Themes were identified and all members of the research team agreed on the final themes. This synthesis method was chosen due to the wide variety of measures and variables reported by the studies.

## 3. Results

**Figure 1:**
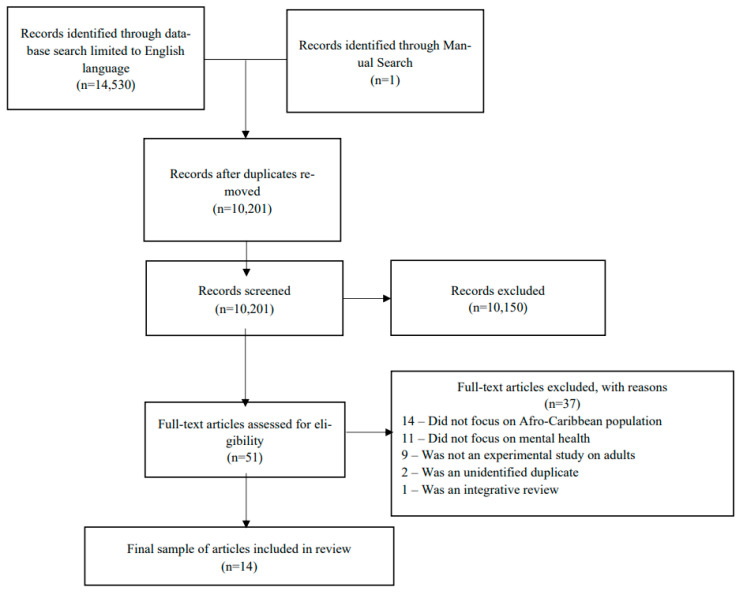
Prisma Flow Sheet.

The search strategy yielded 14,530 potentially relevant reports, of which 4329 were duplicates, leaving a remainder of 10,201 studies. Initial screening using the inclusion and exclusion criteria led to the removal of 10,186 articles. Ineligible papers were most often eliminated for not focusing on the African Caribbean population. An additional study was added after the initial search due to its later publication, and inclusion and exclusion criteria were applied to this study manually to confirm eligibility. The final sample included 14 articles between 2006 and 2021 (Figure 1).

The included studies were observational, cross-sectional, and one qualitative. The number of study participants ranged from 57 to 6082. Most of the studies were conducted in the US and focused on a variety of NCDs (e.g., diabetes, hypertension, cardiovascular disease). Study characteristics and key findings are described in Table 1. None of the selected studies were rated as being poor in quality. The findings of the quality assessment are summarized in Table 2 and Table 3.

Clinical, anthropometric, psychological, and biometric measures were studied in most articles. Clinical variables included blood pressure, height, weight, waist circumference, and body mass index; laboratory variables included low-density lipoprotein, total cholesterol, hemoglobin A1c (HbA1c); self-reported variables included medication adherence, anxiety, stress, depression, history of stroke, hypertension, and/or diabetes. Instruments used included the Perceived Stress Scale (stress), Beck Depression Inventory Scale (depression), Morisky Medication Adherence Scale (medication adherence), level of antidepressant use (medication utilization), and World Health Organization’s Composite International Diagnostic Instrument (mental disorder). Additional addressed variables included physical activity, education, language preference, social support, socioeconomic status, smoking status, and education level.

### 3.1. Data Synthesis

Three synthesized themes were identified: (1) prevalence of comorbid mental health problems and chronic NCDs (*n* = 10); [20,21,22,23,24,25,26,27,28,29]; (2) factors that mitigate or mediate the association between mental health problems and chronic NCDs (*n* = 11) [23,24,25,26,27,28,30,31,32,33,34]; —(2a) factors influencing self-management (*n* = 4); [26,27,29,30]; (2b) association between mental health and NCD outcomes—studies focused on (2b1) risk factors (*n* = 9) [23,24,25,26,27,28,31,32,33]; and (2b2) protective factors (*n* = 3); [24,31,33]; and (3) varied results (*n* = 3) [27,28,29]. The results are summarized in Table 4.

#### 3.1.1. Comorbid Mental Health Problems and Chronic NCDs

Comorbid poor mental health and chronic NCDs were reported throughout the literature, with 7 out of 14 studies reporting a statistically significant association between mental health problems (e.g., depression) and comorbid NCDs in African Caribbeans [21,22,23,24,25,26,30]. Stewart et al. reported a strong association between depression and physical illness and subsequent disability (e.g., stroke) in the African Caribbean population (OR = 2.48) [20]. These results were mirrored by Watkins et al., as they revealed that the odds of having more than one comorbid chronic NCD were greater for African Caribbeans with major depressive disorder when compared to those without (OR = 3.66) [21]. Similarly, Seecheran et al. outlined how the number of comorbid conditions in African Caribbean populations was positively correlated with major depressive disorder symptoms (OR = 1.125) [22]. Frederick and Maharajah explored the relationship between mental health measures and type 2 diabetes [23]. They found African Caribbeans with coexisting medical complications and type 2 diabetes reported more incidents of severe depression, with a Zung Depression Scale average score of 6.27 higher than those with type 2 diabetes alone [23]. Additionally, Frederick and Maharajh highlighted a correlation between coexisting medical conditions and depression (*r* = −0.32) [23].

Comparably, Huffman and associates [24] identified that African Caribbeans with type 2 diabetes had a depression score 3.2 points higher than those without type 2 diabetes. de Caluwé et al. showed diabetes prevalence to be 7.6% higher in patients with significant mental illness than in the general population [25]. Among patients with diabetes and depression, 11% had a heart condition and 15% had hypertension [23]. de Caluwé et al. reported that the prevalence of hypertension was statistically significantly higher (37.4%) in all patients with significant mental illness compared to the general African Caribbean population (19.9%) [25]. In a large study of African Caribbeans (*n* = 1203), 80% had comorbid depressive symptoms, with 25% having severe depressive symptoms [22]. The same study found that cardiovascular disease, hypertension, chronic kidney disease, and chronic obstructive pulmonary disease were statistically significantly associated with depressive symptoms (OR = 1.988) [22]. Conversely, Sims and colleagues associated optimism with ideal blood pressure (*p* > 0.05), depression, and major life events contributing to obesity prevalence and CVD conditions [26]. The same study found that mental health measures, including cynicism, negative affect, stress, and major life events, were associated with obesity prevalence (*p* < 0.05) [26]. Stewart et al. identified an association between depression and previous stroke history in the United Kingdom (UK) population born in the Caribbean (OR = 4.93) [20]. Assari and Lankarani, observed an association between heart disease and depressive symptoms in African Caribbeans, unlike other Blacks or non-Hispanic Whites living in the US (OR = 18.174) [28]. Collins-McNeil calculated 10% of the variance in cardiovascular risk to be accounted for by anxiety, depression, and perceived social support scores (*R*^2^ = 0.095) [29]. Overall, the included studies imply a statistically significant association between mental health disorders, particularly depression, and chronic NCDs, among African Caribbeans.

#### 3.1.2. Factors That Mitigate or Mediate the Association between Mental Health Problems and Chronic NCDs

##### Factors Influencing Self-Management

Self-management of NCDs in African Caribbeans was influenced by several factors, including cultural health beliefs and environment. Sims et al. reported that mental health measures such as high anger and stress reduced diabetes control (*p* > 0.05) [27]. Three studies clarified the relationship between social support or isolation and self-management of diabetes and mental health disorders, including depression and anxiety [26,29,30]. Collins-McNeil identified social support to be negatively correlated with the trait (*r* = −0.505) and state anxiety (*r* = −0.511) as well as depressive symptoms (*r* = −0.479) [29]. In a 2021 qualitative study, Magny-Normilus and colleagues highlighted social isolation as a contributing factor inhibiting the ability of older African Caribbeans to manage their type 2 diabetes [30].

##### Association between Mental Health and NCD Outcomes

Risk Factors

Social determinants of health were found to influence the level of care for NCDs. Magny-Normilus et al. identified diabetes as “not a poor man’s disease” due to the self-management requirements of food, insulin, and doctor visits [30]. They highlighted the lack of resources as a barrier to self-management. Substance use has been tied to mental illness in the literature. Seecheran et al. found African Caribbean individuals to have elevated levels of substance use disorder (SUD) when significant mental illness was present [22]. The same study revealed an association between metabolic syndrome and SUD (*p* < 0.001) [22]. Frederick and Maharajh showed a positive correlation between the number of substances used and having major depressive disorder (OR = 1.062) and NCDs, where hypertension and type 2 diabetes were the two most common coexisting medical conditions [23].

Sex was reported as a possible factor affecting the association between NCDs and mental health. For example, many physical health comorbidities were more prevalent in African Caribbean women than men [25,27,31]. De Caluwé found that female patients with significant mental health illnesses had a higher prevalence of diabetes (28.2%), obesity (50.0%), and increased waist circumference (88%) when compared to men [25]. Frederick and Maharajh outlined that African-Caribbean women with type 2 diabetes had a 10.4% increased likelihood of depression compared to their male counterparts [23]. Sex differences also affected self-management. Sims et al. strongly associated psychosocial factors in women with lower NCD control, but this was not found in men. The same study identified that women with high cynicism had a 38% lower prevalence of hypertension control [27].

Protective Factors-Some biological measures were proposed to aid NCD management [24,31,33]. Overall, Fuster’s team reported Haitian-born residents in Chile to be generally healthy, with a lower prevalence of obesity and cardiovascular risk than other residents of Chile [31]. Young et al. determined that African Caribbeans had average problem areas, in a diabetes score that was 10 points lower when compared to European American and Asian Indian participants [33]. It was common among studies to report reduced substance rates among African Caribbeans. Fuster and colleagues found African Caribbeans to have low levels of alcohol use, with 7.3% of individuals drinking more than once a week and 4.2% of individuals smoking [31]. Huffman et al. reported African Americans to have a 40% prevalence of smoking, while African Caribbeans had a 6% prevalence [24].

#### 3.1.3. Varied Results

Three studies reported results that differed from the majority of the data [27,28,31]. Assari and Lankarani associated hypertension with a lack of depressive symptoms [28]. Sims et al. reported the influence of cumulative stress and negative affect on hypertension control among men and the consequence of significant life events on hypertension control in men and women [27]. Fuster et al. described African Caribbeans as having high sedentarism levels (87.9%). Although these studies showed differing results, they were rated good in the quality assessment [31].

## 4. Discussion

This review aimed to further our understanding of the prevalence of mental health problems among African Caribbean adults with chronic NCDs and explore the association between mental health disorders and the self-management of their diseases. This review revealed a strong association between depression and chronic NCDs in the African Caribbean population [20,21,23]. Diabetes and hypertension were associated with the presence of depression and depression severity, stress measures, and significant life events; hypertension alone was also associated with severe mental illness and a high negative affect [23,24,25,26,28,29,32]. Other NCDs, including obesity, stroke, cardiovascular disease, heart disease, and atherosclerosis, were also associated with mental health measures such as cynicism, negative affect, stress, major life events, anger, and depressive symptoms [20,27,28]. Ahmed and Conway found that American Indians and Hispanics were more likely to suffer comorbid mental health and NCDs than their White counterparts [11]. These results align with the literature that describes the comorbidity between mental health and chronic NCDs and the variance in comorbidity between ethnic groups [11,35,36].

While the majority of included studies identified an association between NCDs and mental health in the African Caribbean population, three studies reported a lack of association between depression and hypertension, as well as depression and diabetes [22,28,32]. Mental health measures were reported to improve hypertension control in one study (Sims et al., 2020) [27]. These variances are similarly reflected in the broader literature, as a 2015 study by Lankarani and Assari found statistically significant comorbidity between NCDs and depression for European Americans and African Americans, but not African Caribbeans [28]. While most of this study’s results report comorbidity between chronic NCDs and mental health, this variance must still be considered. It may highlight a need for future research to quantify the relationship between chronic NCDs and mental health in populations with varying racial and ethnic backgrounds.

Comorbidity between NCDs and mental health problems is important to recognize because it influences hypertension and diabetes control in the African Caribbean population [22,27]. Mental health challenges have also been shown to reduce the practice and success of self-management behaviors in other populations [16,37]. Axon et al. found an association between medication adherence and depression in patients with type 2 diabetes for non-Hispanic Blacks, Hispanics, non-Hispanic Whites, and people of other racial/ethnic groups [16]. Nanayakkara et al. supported these results in an Australian population [38].

Social determinants of health and substance use were found to negatively impact the self-management of chronic NCDs in the African Caribbean population [21,24,30]. The results of this review are supported by Walker et al. and their findings of a direct relationship between glycemic management in subjects with type 2 diabetes and psychosocial determinants of health [39]. While, overall, African Caribbean individuals had reduced substance use [24,31], those with severe mental illness were found to have elevated substance use disorder [24].

Within the African Caribbean population, sex played a role in self-management, mental health, and physical health measures. Biological sex, rather than gender, was analyzed for this review, as it reflects the previous body of research. Further research is needed on the relationship between gender and self-management, mental health, and physical health measures in the African Caribbean population. African Caribbean women were found to have more poorly controlled NCDs than men, with a higher rate of metabolic syndrome, type 2 diabetes, body mass index, obesity, and waist circumference [25,31]. The increase in weight and associated conditions for African Caribbean women may stem from ingrained cultural preferences and practices that shapely framed Black women are more attractive in their communities. A 2019 study found that African Caribbean women had an aversion to being “too thin” rather than “too thick,” which paralleled the beliefs of African American women [40].

This study reveals women have higher rates of depression, the stress of all categories, and diagnosis of affective disorders [22,23,24,25,27,32]. On the other hand, men were more likely to be diagnosed with schizophrenia, psychotic disorders, low optimism, cynicism, and substance use disorder [25,27]. The difference in diagnosis and increased substance use in men may be attributed to cultural views of mental health. Smith et al. summarized the stigma against men’s mental health in 2018, and they highlighted that men were less likely to seek help for mental health problems, less likely to report symptoms, and more likely to experience substance use that significantly affects their lives as women [26]. These findings may negatively impact chronic disease management and could be associated with the higher rates of cardiovascular conditions in African Caribbeans. 

Aside from factors that reduce self-management outcomes, family and general social support were found to improve self-management for diabetes and mental health conditions [29,30]. The African Caribbean population reported better self-management strategies generally, consuming fewer calories, following dietary advice, practicing healthier eating, and having fewer problem areas in diabetes management than other populations [30,33]. The study by Fuster et al. varied in its results on self-management and reported high levels of sedentary lifestyles in the African Caribbean population [31]. The protective social factors and self-management practices may result in improved biological measures in the African Caribbean population, such as decreased waist circumference, decreased low-density lipoprotein, decreased instances of obesity, and lower body mass indexes [30,31,41].

### Limitations

Only English language publications were analyzed. Restricting the review to one language may have omitted other relevant studies unique to this ethnic group. Our focus was on the African Caribbean population. Immigration status was not considered in this review. Broader populations were considered applicable in this study because of the limited number of studies specifically focusing on African Caribbeans. This is especially true for African Americans, a demographic that African Caribbeans are often included in but not often differentiated from. As a result, this review is a global representation of African Caribbeans deriving from various unique backgrounds and environments. 

## 5. Conclusions

The African Caribbean population is at elevated risk of NCDs, mental health disorders, and comorbid conditions. This population has unique factors that influence self-management and health outcomes, including social determinants of health, high substance use in those with significant mental illness, sex differences, social support, and reported high levels of self-management adherence. Despite the wealth of knowledge surrounding the high prevalence and comorbidity of NCDs and mental health in the African Caribbean population, there is limited research on how mental health influences self-management of NCDs in the African Caribbean population specifically. Additionally, despite, the abundance of evidence on the prevalence of mental health disorders and NCDs, this analysis indicates a paucity of intervention research to improve mental health and management of NCDs. Thus, future research should quantify and qualify the relationship between mental health and NCD self-management in the African Caribbean population. This research is needed to inform strategies to effectively address comorbid mental health problems and NCDs in the African Caribbean population in the US.

## Figures and Tables

**Table 1:** Study Characteristics and Key Findings.

Author, Year	Purpose	Design	Location/ Country	Sample	Age Range	Ethnicity	Measures	Key Findings
[20]	To examine the association between stroke, vascular risk factors, and depression	Cross-sectional	South London	287	55–75	African Caribbean	Stroke, HTN, T2D, angina, height, weight, waist to hip, smoking, and physical activity	A strong association between depression and physical illness and subsequent disablement (OR = 2.48). An association between depression and previous stroke history (OR = 4.93).
[21]	To test whether differences between race and ethnic groups exist for major depressive disorder (MDD) and generalized anxiety disorder (GAD) with one or more chronic medical conditions	Cross-sectional	US	5889	18+	African American, African Caribbean, and European American	Socio-economic status, MDD, GAD, and chronic medical conditions	The odds of having multiple comorbid conditions were greater for African Caribbeans with MDD when compared to those without (OR = 3.66).
[22]	To determine any associations among patient demographics, comorbidities, and cardiovascular/depressive symptoms	Cross-sectional	Trinidad and Tobago	1203	18+	South Asian, African Caribbean, Multiracial/Other	HTN, cerebro-vascular events, kidney disease, pulmonary disease, and depression	The number of comorbid conditions in African Caribbean populations was positively correlated with MDD symptoms (OR = 1.125). 40% of participants with cardiovascular disease had comorbid depression, and 25% displayed significant depressive symptoms. Cardiovascular disease, hypertension, chronic kidney disease, and chronic obstructive pulmonary disease were significantly associated with depressive symptoms (OR = 1.988). Women had a 1.7-fold incidence and prevalence of depression. Depressive symptoms were not significantly associated with the clinical parameters of diabetes. African Caribbean individuals had elevated levels of substance use disorder when significant mental illness was present. An association between metabolic syndrome and SUD (*p* < 0.001).
[23]	To describe the statistical prevalence of depression in type 2 diabetes (T2D)	Cross-sectional	Trinidad and Tobago	128	21+	Indo-Trinidadian	Socioeconomic status, glucose control, medical complications, and depression	A correlation between coexisting medical conditions and depression (*r* = −0.32). Caribbean Blacks with coexisting medical complications in addition to T2D reported more severe depression, with a Zung Depression Scale average score of 6.27 higher than those with T2D alone. Heart conditions (11%) and hypertension (15%) were the two most common coexisting medical conditions with T2D and depression in Caribbean Blacks. Positive correlation between the number of substances used and having MDD (OR = 1.062). Caribbean black women with T2D had a 10.4% increased likelihood of depression compared to their male counterparts.
[24]	To assess associations between depressive symptoms and perceived stress with beta-cell function	Cross-sectional	Miami, Dade, and Broward CountiesFlorida/US	462,696	18+	African and Haitian Americans	Fasting plasma glucose, weight, height, waist circumference, blood pressure, lipid panel, serum insulin concentration, Beck depression inventory, depression, and stress	Caribbean Blacks with T2D had a depression score 3.2 points higher than those without T2D. African Caribbeans had a lower beta-cell function (42.2 HOMA2), higher fasting plasma glucose (162.6 mg/dL), HbA1c (8.5%), and higher depression score (10.9) compared to African Americans (63.5 HOMA2, 147.3 mg/dL, 7.6%, 9.5). African Americans had a 40% prevalence of smoking, while African-Caribbeans had a 6% prevalence.
[25]	To determine the prevalence of metabolic syndrome in patients with severe mental illness	Cross-sectional	Island of Curacao	350	18–84	African Caribbean	Cardiovascular disease, HTN, hyper-glycemia, T2D, obesity, total cholesterol, metabolic syndrome, psychiatric diagnoses, and substance disorder and use	Diabetes prevalence was 7.6% higher in patients with significant mental illness compared to the general population. The prevalence of hypertension was significantly higher (37.4%) in all patients with significant mental illness compared to the general African Caribbean population. Women had a 38.2% higher prevalence of metabolic syndrome than men. Women had a higher prevalence of increased waist circumference (51%), low HDL (50.6%), hypertension (49.4%), hyperglycemia (28.6%) compared to men. Women with significant mental illness had a higher prevalence of diabetes (28.2%), obesity (50.0%), and increased waist circumference (88.2%) compared to men. Male participants had a 44.8% elevated prevalence of substance use compared to women. Men were more likely to be diagnosed with schizophrenia and related psychotic disorders compared to their female counterparts (*x*^2^(2) = 6.03).
[26]	To examine the associations between positive optimistic orientation and LS7 among African Americans	Cross-sectional	Jackson, MS/US	4734	35–85	African American	Optimism, LS7 components, demographics, socioeconomic status, depression HTN, HLD, and blood glucose	Optimism was associated with ideal blood pressure (*p* > 0.05).
[27]	To demonstrate the cumulative effects of individual psychosocial factors and CVD risk factors by sex	Cross-sectional	Jackson, MS/US	4806	35–84	African American	Cynicism, anger, depression, global and weekly stress, major life events, cardiovascular disease, and risk factors	High negative affect was associated with hypertension (*p* < 0.05). Cynicism, negative affect, stress, and major life events were associated with obesity prevalence (*p* < 0.05). High anger and stress reduced diabetes control (*p* > 0.05). Women had a higher prevalence of both obesity and hypertension than men (*p* < 0.05). Women had greater depressive symptoms and overall stress than men (*p* < 0.05). Psychosocial factors were strongly associated with lower NCD control in women, but not in men. Women with high cynicism had a 38% lower prevalence of hypertension control. Psychosocial factors were more strongly associated with hypertension and diabetes prevalence for men than women. Men who reported high cynicism had a 12% increased prevalence of hypertension compared to women. Cumulative stress and negative affect improved hypertension control among men, and major life events improved hypertension control in men and women.
[28]	To compare differing racial/ethnic populations for associations between NCDs and medical comorbidities	Cross-sectional	US	6082	18+	African American, African Caribbean, European American	Demographics, NCDs, medical comorbidities, GAD, and MDD	Heart disease and depressive symptoms were associated in African Caribbeans unlike African Americans or European Americans (OR = 18.174). Hypertension was associated with a lack of depressive symptoms.
[29]	To examine the ability for depression, anxiety, and social support to predict cardiovascular disease risk in those with no previous cardiovascular events	Cross-sectional	Southern US	57	35–74	African American	Social support, cardiovascular disease risk, depression, and anxiety	10% of the variance in cardiovascular risk accounted for anxiety, depression, and perceived social support scores (*R*^2^ = 0.095). Social support was negatively correlated with trait (*r* = −0.505) and state anxiety (*r* = −0.511) as well as depressive symptoms (*r* = −0.479).
[30]	To describe the experiences of older adult Haitian Immigrants in managing T2D	Qualitative, Observational	Northern US	20	65+	Haitian Immigrants	HbA1c, HTN, emotions, culture, and education	Social isolation was a major factor inhibiting the ability of older African Caribbeans to manage their T2D. Diabetes was considered “not a poor man’s disease” due to the self-management requirements of food, insulin, and doctor visits, and the lack of resources was highlighted as a barrier to self-management.
[31]	To acquire the basic health information of the Haitian adult population living in Chile	Cross-sectional	Chile	499	18+	Haitian born immigrants in Chile	BMI, blood pressure, lipids, nutritional status, diabetes, substance use, quality of life, physical activity, mood, and depression, and renal function	Women had higher proportional obesity, and abdominal circumference compared to men. Described African Caribbeans to be generally healthy, with a lower prevalence of obesity and cardiovascular risk. African Caribbeans had low levels of alcohol use with 7.3% of individuals drinking more than once a week, and 4.2% of individuals smoking. African Caribbeans had high levels of sedentarism (87.9%).
[32]	To assess the prevalence and correlation of depression and T2D self-management and control	Cross-sectional	Northern Manhattan/US	360	35–70	Hispanics of Caribbean Origin	Depression, antidepressant use, stressful life events, education, HbA1c, and medication adherence.	A strong association between female sex, and clinical depression (OR = 2.30). Depressive symptoms were not significantly associated with clinical parameters of diabetes.
[33]	To assess associations between diabetes- related stress and predicted cardiovascular risks and complications.	Cross-Sectional	East Oakland, US	48	40–80	European American, African Caribbean, Asian-Indian	PAID scores, HbA1c, HDL, LDL, Afib, BP, and smoking status	The average man’s problem areas in diabetes score was 2.9 points higher than the average woman’s. African Caribbeans had average problem areas in diabetes score 10 points lower than European American and Asian Indian participants.

**Table 2:** National Institutes of Health Risk of Bias Assessment.

Study	[20]	[32]	[24]	[31]	[25]	[29]	[27]	[28]	[23]	[21]	[23]	[22]	[26]
Was the research question or objective in this paper clearly stated?	Y	Y	Y	Y	Y	Y	Y	Y	Y	Y	Y	Y	Y
Was the study population clearly specified and defined?	Y	Y	Y	Y	Y	Y	Y	Y	Y	Y	Y	Y	Y
Was the participation rate of eligible persons at least 50%?	Y	Y	NR	Y	NR	NR	NR	Y	Y	NR	Y	Y	NR
Were all the subjects selected or recruited from the same or similar populations (including the same time period)? Were inclusion and exclusion criteria for being in the study prespecified and applied uniformly to all participants?	Y	Y	N	Y	Y	Y	Y	Y	Y	Y	Y	Y	Y
Was a sample size justification, power description, or variance and effect estimates provided?	Y	Y	N	Y	Y	Y	NA	Y	Y	Y	Y	Y	Y
For the analyses in this paper, were the exposure(s) of interest measured prior to the outcome(s) being measured?	Y	Y	Y	Y	Y	Y	NA	Y	Y	Y	Y	Y	NA
Was the timeframe sufficient so that one could reasonably expect to see an association between exposure and outcome if it existed?	Y	NA	NA	Y	NA	NA	NA	Y	Y	Y	NA	Y	NA
For exposures that can vary in amount or level, did the study examine different levels of the exposure as related to the outcome (e.g., categories of exposure, or exposure measured as continuous variable)?	NA	Y	Y	NA	Y	N	Y	NA	NA	NA	NA	NA	NA
Were the exposure measures (independent variables) clearly defined, valid, reliable, and implemented consistently across all study participants?	NA	Y	Y	Y	Y	Y	Y	NA	NA	NA	NA	NA	Y
Was the exposure(s) assessed more than once over time?	N	N	N	NA	N	N	N	N	N	N	N	N	N
Were the outcome measures (dependent variables) clearly defined, valid, reliable, and implemented consistently across all study participants?	Y	Y	Y	Y	Y	Y	Y	Y	Y	Y	Y	Y	Y
Were the outcome assessors blinded to the exposure status of participants?	NA	NA	NA	NA	NA	NA	NA	NA	NA	NA	NA	NA	NA
Was loss to follow-up after baseline 20% or less?	Y	Y	NR	Y	Y	NR	NA	Y	Y	Y	Y	Y	NA
Were key potential confounding variables measured and adjusted statistically for their impact on the relationship between exposure(s) and outcome(s)?	NA	Y	Y	NA	Y	Y	Y	Y	Y	Y	Y	Y	Y
Quality Score (Good, Fair, or Poor)	Good	Good	Fair	Good	Good	Good	Good	Good	Good	Good	Good	Good	Good

Available at: https://www.nhlbi.nih.gov/health-topics/study-quality-assessment-tools (accessed on 11 August 2022). Y, Yes; N, No; NA, not applicable; NR, not reported.

**Table 3:** Consolidated Criteria for Reporting Qualitative Research (COREQ).

No.	Item	Guide Questions	Description
**Domain 1: Research team and reflexivity**			
*Personal characteristics*			
1	Interviewer/facilitator	Which author/s conducted the interview or focus group?	CMN
2	Credentials	What were the researcher’s credentials?, e.g., PhD, MD	PhD
3	Occupation	What was their occupation at the time of the study?	Research Scholar
4	Gender	Was the researcher male or female?	F
5	Experience and training	What experience or training did the researcher have?	Previous experience in qualitative research
*Relationship with participants*			
6	Relationship established	Was a relationship established prior to study commencement?	No
7	Participant knowledge of the interviewer	What did the participants know about the researcher?, e.g., personal goals, reasons for doing the research	Unknown
8	Interviewer characteristics	What characteristics were reported about the interviewer/facilitator?, e.g., Bias, assumptions, reasons and interests in the research topic.	None
**Domain 2: Study Design**			
*Theoretical framework*			
9	Methodological orientation and theory	What methodological orientation was stated to underpin the study?, e.g., grounded theory, discourse analysis, ethnography, phenomenology, content analysis	Descriptive data analysis
*Participant selection*			
10	Sampling	How were participants selected?, e.g., purposive, convenience, consecutive, snowball	Recruitment through flyers and meeting inclusion criteria.
11	Method of approach	How were participants approached?, e.g., face-to-face, telephone, mail, email	Flyers at local churches.
12	Sample size	How many participants were in the study?	20
13	Non-participation	How many people refused to participate or dropped out? Reasons?	unknown
*Setting*			
14	Setting of data collection	Where was the data collected?, e.g., home, clinic, workplace	Face to face
15	Presence of nonparticipants	Was anyone else present besides the participants and researchers?	Unknown
16	Description of sample	What are the important characteristics of the sample?, e.g., demographic data, date	Haitian adults aged 65 or older, type 2 diabetes for at least one year, living in the US.
*Data collection*			
17	Interview guide	Were questions, prompts, guides provided by the authors? Was it pilot tested?	Yes
18	Repeat interviews	Were repeat interviews carried out? If yes, how many?	No
19	Audio/visual recording	Did the research use audio or visual recording to collect the data?	Yes, audio recordings
20	Field notes	Were field notes made during and/or after the interview or focus group?	Yes
21	Duration	What was the duration of the inter views or focus group?	40–90 min
22	Data saturation	Was data saturation discussed?	Yes
23	Transcripts returned	Were transcripts returned to participants for comment and/or correction?	No
**Domain 3: Analysis and findings**			
*Data analysis*			
24	Number of data coders	How many data coders coded the data?	2
25	Description of the coding tree	Did authors provide a description of the coding tree?	Yes
26	Derivation of themes	Were themes identified in advance or derived from the data?	Themes were derived from the data
27	Software	What software, if applicable, was used to manage the data?	NVivo 12 software IBM SPSS Statistics for Windows
28	Participant checking	Did participants provide feedback on the findings?	No
*Reporting*			
29	Quotations presented	Were participant quotations presented to illustrate the themes/findings? Was each quotation identified?, e.g., participant number	Yes, quotations were presented and identified
30	Data and findings consistent	Was there consistency between the data presented and the findings?	Yes
31	Clarity of major themes	Were major themes clearly presented in the findings?	Yes
32	Clarity of minor themes	Is there a description of diverse cases or discussion of minor themes?	Yes

**Table 4:** Results Summary.

Themes	Supporting Studies
1. Prevalence of comorbidity mental health problems and chronic noncommunicable diseases	[20,21,22,23,24,25,26,27,28,29]
2. Factors that mitigate or mediate the association between mental health problems and chronic noncommunicable diseases	[23,24,25,26,27,28,30,31,32,33,34]
2a. Factors influencing self-management	[26,27,29,30]
2b. Association between mental health and noncommunicable disease outcomes	[22,23,24,25,26,27,28,29,30,32,33]
2b1. Risk Factors	[23,24,25,26,27,28,31,32,33]
2b2. Protective Factors	[24,31,33]
3. Varied results	[27,28,29]

## Data Availability

Not applicable.
